# The Transformative Influence of La Varenne's *Le Cuisinier Francois* (1651) on French Culinary Practice

**DOI:** 10.3389/fnut.2020.00042

**Published:** 2020-04-28

**Authors:** Leon G. Fine

**Affiliations:** Program in the History of Medicine, Department of Biomedical Sciences, Cedars-Sinai Medical Center, Los Angeles, CA, United States

**Keywords:** La Varenne, French culinary practice, gastronomy, history, cooking

## Abstract

The current proliferation of modern cookbooks targeted to the public at large makes it impossible to conceive of there being any that could have had an overriding influence on culinary practice or eating preferences, even at a local level. However, when there was a historical absence of cookbooks for a half-century, as there was in France in the first half of the seventeenth century, it is argued herein that the advent of a single cookbook in 1651, *Le Cuisinier Francois* by La Varenne, could have had a transformational influence on culinary practice over the ensuing half-century. The book went into more than 50 subsequent editions in the second half of the century. La Varenne stated clearly that his intent was to provide a guide for professional cooks. However, it is hypothesized in this article that the widespread and enduring success of the book was due to its attraction to and acquisition by the general public, including household cooks. This can be ascribed to (i) the fact that there had been no French cookbook describing novel culinary approaches in the preceding 50 years, (ii) La Varenne's concise, uncomplicated, and practical style of presentation of recipes, and (iii) his selection of principal ingredients, which were within the reach of the household cook and which reflected the availability of foods at the time of writing. Furthermore, because *Le Cuisinier Francois* was laid out according to widely observed religious practices, finding the best options for the appropriate day of the month became an easy task for the user. La Varenne initiated a departure from an earlier style of heavily spiced cooking to one that was based on natural flavors, a limited use of spices, and uncomplicated cooking methods. Thus, rather than assuming that the enduring popularity of the book was due to its widespread use by culinary professionals, it is argued that its style and substance must have imparted a sense of empowerment and confidence in the home cook and that, in these terms, La Varenne's influence on culinary practice was far more widespread and truly transformative, accounting for the remarkable success of *Le Cuisinier Francois*.

## Introduction

It is rare that a single publication can be shown to have influenced the historical course of a field of endeavor. Examples of this in physiology and medicine in the sixteenth and seventeenth centuries are Vesalius's *De Humani corporis fabrica libri septem* (1543), describing and illustrating human anatomy, and Harvey's *De Motu Cordis* (1628), describing for the first time the circulation of the blood (1).

In the field of French culinary and gastronomic creativity, the beginnings of such singular influences emerged. In his comprehensive survey of cookbooks from 1470 to 1700, Notaker finds a paucity of French cookbooks prior to the midpoint of the seventeenth century (2). Starting with the famed publication by Taillevant (1486), which went into 21 editions, and that of Platina (1505), which reached 16 editions in the same period, Notaker identifies only four cookbook publications, described below, in the first half of the seventeenth century (3).

The vast majority of French books of sustained influence published in the 1700s and appeared in the second half of the century. So, is it at all reasonable to argue that a single publication could have had a transformative influence on the course of French culinary practice during this period? This paper argues that this was, indeed, the case, and points out that the style and content of a single cookbook managed to influence culinary practice by non-professional and home cooks in France for at least a half-century. The book was *Le Cuisinier Francois*, published in Paris in 1651, and the author, was Francois Pierre, *nom-de-plume* La Varenne.

## A Hypothesis

This paper does not claim to make the original case that La Varenne was an innovator and a major figure in transforming the direction of French cuisine in the seventeenth century. This case has been ably made by others (3–5). Herein, however, I ask, why and how did this come to pass? After all, this was just a single, pocket-sized cookbook, and reading and consulting with such books were not exactly central in the minds of the French public of the times (3). How did this publication come to be so influential in mid-seventeenth century France?

It is my hypothesis that the popularity and influence of La Varenne's publication can be best measured by the multiple editions through which it went, in France and beyond. This could only have been due to its widespread adoption by the French public at large, rather than by the professional chefs at whom it was initially aimed.

For this hypothesis to be substantiated, the following understandings would need to apply:

1. In mid- seventeenth century France, the public was particularly receptive to instruction on how to be creative in the kitchen because an innovative cookbook had not been published in the half-century prior to the publication of *Le Cuisinier Francois*.
2. La Varenne based his recipes on foods widely available to the French public at this time.
3. The recipes in *Le Cuisinier Francois* were practical enough to be adopted and executed by home cooks and caterers, and were presented in a precise and understandable style. This enabled translation from recipe to table at a time the public was already moving toward a lighter and healthier style of cuisine.

The following sections examine the basis for each of these contentions:

### The Cook

**Figure 1:**
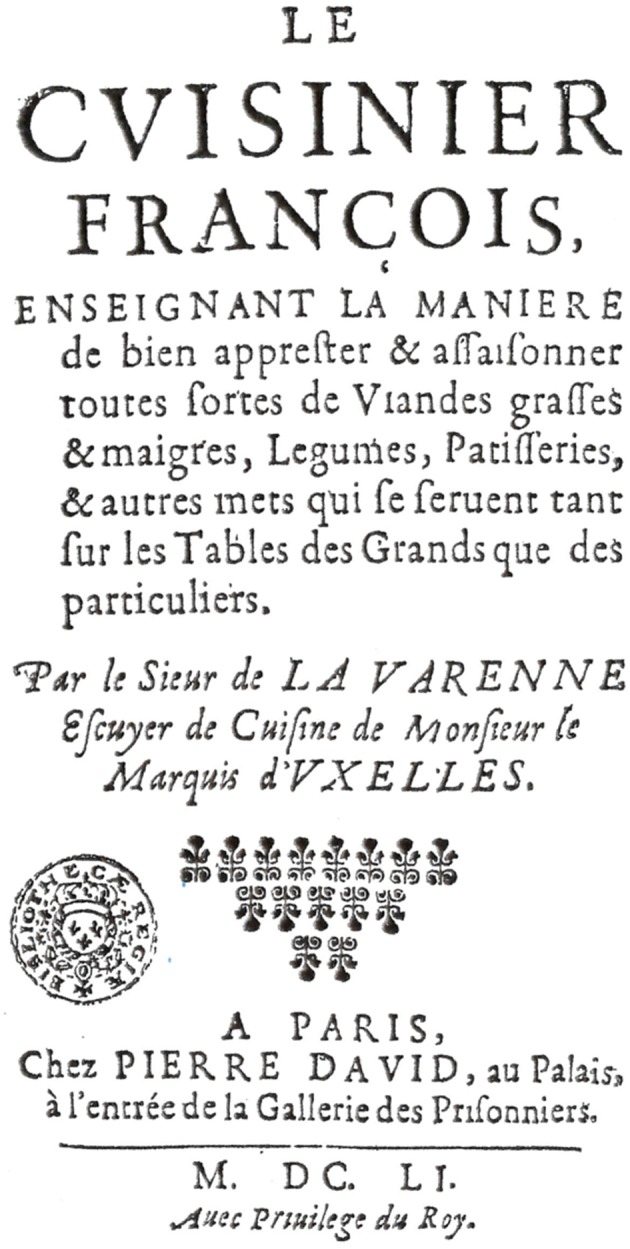
Title Page of first edition of *Le Cuisiner Francois* (1651).

The mid-seventeenth century serves as a convenient starting point. Whether by chance or as a consequence of events of the time, a cookbook, *Le Cuisinier Francois*, authored by Francois Pierre, *nom de plume* La Varenne, was published in France in 1651 (6) (Figure 1). La Varenne was born in Chalone-sur-Saone in Burgundy in 1615, and lived in Dijon for 60 years until his death in 1678 (Pinkard notes that this *nom de plume* linked him to an ennobled cook, Guilleme Fouquet, who was rewarded by his master, Henry IV, with the title Marquis de la Varenne) (4).

Around the age of 25, he entered into the service of the Marquis d'Uxelles (Louis Chalon du Ble, died 1658) as a cook, where he was employed at the time of publication of his book. During his decade of service, he “found the secret how to make meates (sic) ready, neatly and daintily” and was able to exercise his emerging talent to the benefit of the French princes and marshals, with whom the Marquis associated. He dedicated the book to the Marquis, assigning to himself the designation “clerk of your kitchin (sic),” a token of his passion for his service to his generous master.

Little more is known about the personal life of La Varenne. He was a member of the fraternity of cooks of the renaissance, about whom he states: “Of all the cookes (sic) in the world, the French are esteemed the best, and of all the cookes that ever France bred up, this may well challenge the first place, as the nearest and compleatest (sic) that ever did attend the French Court and Armies” (7). This rather self-congratulatory introduction was not off the mark since, French cooks in the seventeenth century were already held in the highest regard due to the innovative food emerging from their kitchens.

It is worth considering the matter of La Varenne's creativity in light of the following statement by Philip and Mary Hyman in their introduction to the reprint of the 1653 English translation of *Le Cuisinier Francois* (7): “La Varenne cannot be credited with inventing the new cuisine presented in his book.” They go on to point to his dedicatory letter in the first edition, in which he states (referring to his master): “I have found the secret how to make meats ready, neatly and daintily… I think that the public ought to receive the profit of this experience of mine, to that end it may owe unto you all the utility which it will receive thereby. I have therefore set down in writing what I so long practiced in the honor of your service.” They appear to conclude from these remarks that La Varenne did not innovate his creations, but acquired them “in the course of his employ.” They go on to declare that “his great contribution to French cuisine—no small accomplishment indeed—is to have been the first to set them down in writing.”

It is my belief that this conclusion fails to recognize the common practice of the times, when a (sometimes sycophantic) profusion of gratitude and thanks was conferred upon a master or patron as a matter of common practice, written in an established style in a dedicatory letter, and in which exaggerated flattery was the norm. Naturally, La Varenne must have acquired his expertise and developed his methods during the period of his employment of over a decade in a well-resourced kitchen, learning from experience and from experimentation, as would be expected of anyone with a sense of having something of value to impart. He surely must have learned from colleagues in the kitchen, but it is difficult to accept that he learned his “culinary secrets” and creations from a master whose nobility would hardly have allowed him to be drawn into the kitchen or, heaven forbid, roll up his sleeves to demonstrate his putative culinary talents to an employee. It is thus my contention that La Varenne should be regarded as a highly original and creative individual and as an originator of an important trend in culinary practice.

La Varenne's lifetime overlapped with that of Louis XIV (1643–1715), a monarch whose eating habits and gourmandise became the substance of legend, and who had often been observed by hundreds as he dined alone in the gardens of the Versailles palace. La Varenne learned to cook in the luxury style expected by the aristocrats of his time. However, it is argued here that his legacy was established by the fact that his methods were succinctly and accurately described in a “cookbook,” which may have been aimed initially at culinary professionals but was ultimately adopted by a wide swath of the French public. This legacy is based upon his transformation of a medieval style of cooking, extant under the aristocracy of his day, into a fundamentally new French style of cooking.

### The Cookbook

La Varenne's publishers, no doubt, had much to profit from this reputation. Realizing its potential significance, an English publisher released an English language version in 1653 (8), based upon the second (revised) French edition of 1652, with a title page that reads: “The French Cook, prescribing the way of making ready all sorts of meats, fish and flesh, with the proper sauces, either to procure appetite or to advance the power of digestion. Also the preparation of all herbs and fruits so as their natural crudities by art opposed; with the whole skill of pastry-work, together with a treatise of conserves both dry and liquid a la mode de France, with an alphabetical table explaining the hard words and other useful tables. Written in French by Monsieur De La Varenne, chef of the kitchen to the Lord Marquess of Uxelles and now Englished by I.D.G. London, May 27. Printed for Charles Adams and are to be sold at his shop at the Sign of the Talbot neere St Dunhams Church in Fleetstreet, 1653.” A reprint of this book was published in 2001 (7).

In Notaker's comprehensive compendium of French cookbooks published between 1470 and 1700 (3), he lists only four cookbooks that were published in France between 1600 and 1650. These were: (1) Olivier de Serres, *Theatre de l'agriculture* (9), which focused upon agricultural products with some advice on food preparation; (2) Lancelot de Casteau, *Ouverture de cuisine* (10), which contained 190 recipes for ordinary, daily dishes and which Notaker judges to have been influenced strongly by other European food traditions; (3) *Le Tresor de sante* (11), a book about foodstuffs, which included recipes; and (4) Philbert Guybert, *Toutes les oeuvres charitables* (12), a collection of recipes for medicines and different healthy drinks, a book that was re-published in about 40 new editions between 1634 and 1670. These publications recapitulated existing culinary approaches without adding anything novel to the preceding half century of cookbooks published in seventeenth century France.

### Sustained Readership of the Cookbook

*Le Cuisinier Francois* was aimed at a readership of culinary professionals, reflective of the dishes La Varenne served to the aristocratic diners for whom he was cooking, but the success of the book was nothing short of astounding. The first edition of 1651 was an instant success, with the second being published the following year and the third, the year after (7). Over the ensuing decade, the book was republished 18 times. By 1700, *Le Cuisinier Francois* had gone through 52 editions (2). Plagiarized versions abounded. For this level of circulation, the book must have found its way into the kitchens of non-professional and home cooks and must have had a substantial influence on the choice of foods and the styles of cooking by the public at large. This influence extended beyond the borders of France, with additional translations in English appearing in 1654 and 1673 and in Swedish in 1664 and 1684 (3).

In his introduction, La Varenne wrote “for my fellows in the profession… of whom some, lacking experience or a ready memory, are unwilling or too timid to become involved in learning what they do not know…” (7). This was an ambitious project since, traditionally, cooks learned their profession by instruction, observation, experimentation, and experience. The advent of print could have been viewed as undermining the professional chef by making culinary knowledge available to anyone who might choose to access it. In France, the trade guilds that regulated professions such as baking, confectionery, and patisserie guarded their secrets jealously.

Davis points out that the term “cookbook” originally referred to a wide range of texts that included lists of ingredients, instructions for preparation of dishes, procedures for food preservation, medical remedies and tonics, and advice on managing a kitchen, organizing meals, and table presentations (13). Ordinary people could now have access to this information. The price of the 1651 edition was 30 to 40 fr (14), which translates into a current price of about 3–4 euros, attesting to the affordability of the book for the home cook. The proliferation of cookbooks subsequent to La Varenne's contribution (3) is testament to such a revolution in the public sphere. Publishers capitalized commercially from this realization.

An important stylistic element was La Varenne's use of terms such as “*methode*” and “*discours*” (method and presentation) in preference to the more traditional terms such as “treatise” and “doctrine,” which would have been used in more traditional scientific literature (3). This stylistic change must have made the book more approachable to the home cook, who would have shied away from treatises with formidable scientific titles.

As Notaker states, La Varenne was “the first person to systematically try to realize this ideal of clarity and understanding. He distinguished between the basic sauces and preparations and the more elaborate ones, and he also gave cross references, so that the book appeared to be a coherent totality codifying the art of cookery” (3).

**Figure 2:**
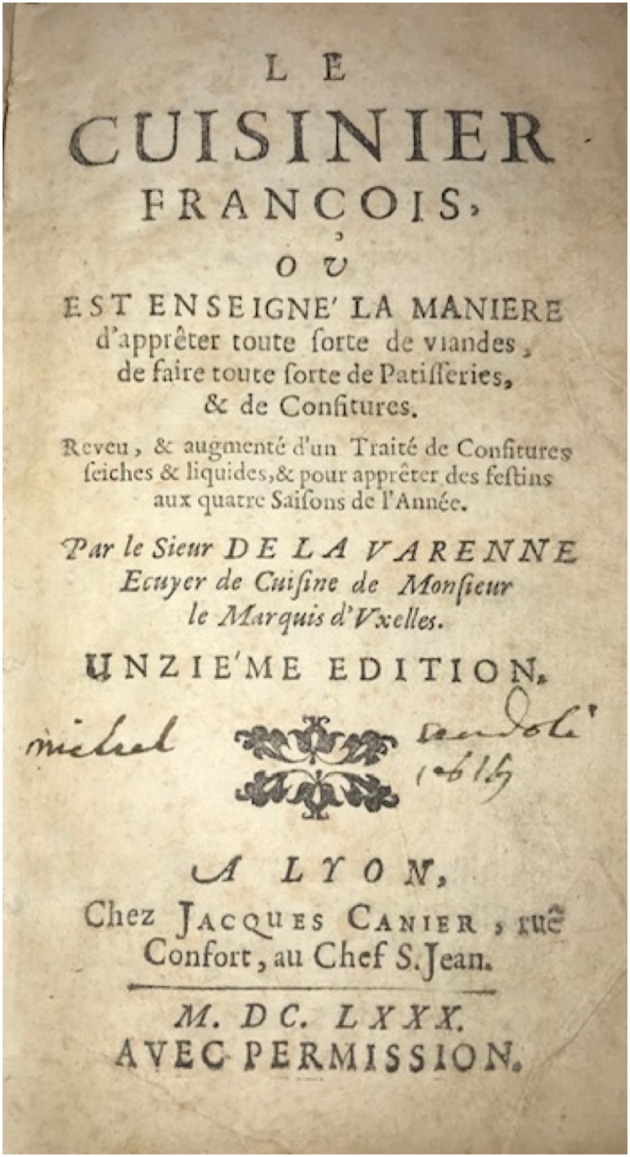
The enduring posthumous reputation of *Le Cuisiner Francois*: Title page of the “eleventh edition” published in Lyon France in 1680. This reads (in English translation): “The French Cook, wherein it is taught how to prepare all sorts of meats, to make all sorts of baked goods and preserves. Reviewed and augmented by a manual of preserves, dry and liquid, to prepare feasts during the four seasons of the year. By Mr. De La Varenne, Chef de Cuisine for Mr Marquess d'Uxelles. Eleventh Edition (published or edited) in Lyon, at Jacques Canier's, rue Confort, Chef St Jean 1680. With authorization.” An octavo volume (5 × 3 inches) containing three sections: (i) *Le Cuisinier Francois*, (ii) *Le Pâtissier Francois*, and (iii) *Le Confiturier Francois*. It is likely that none of the three sections were written by La Varenne himself, the attribution having been added to improve the attractiveness of the book to the buyers who recognized the name and reputation of the presumed author. The pocket-sized book was presumably meant to be used in the kitchen (From the library of the author).

Attesting to the popularity of the book and its value to publishers beyond the lifetime of La Varenne is the insight gained by considering a book published in Lyon in 1680 bearing his name (15). The title page of this 11th edition, printed in Lyon, is shown in Figure 2. The text of the first section, entitled *Le Cuisiner Francois*, bears no resemblance whatsoever to the original texts published in 1651 and 1652. It was bound into a single volume together with previously published sections entitled *Le Patissier Francois* (first published in 1653) and *Le Confiturier Francois* (first published in 1659). Although these latter two sections have often been attributed to La Varenne and bound with his *Le Cuisinier*, Willan disputes this attribution (5), arguing that the lengthy and explicit recipe style are not those which conform to the sketchy style of La Varenne, “who wrote as if he were dictating while stirring a pot*.”* She notes that the style of *Le Patissier* is remarkably similar to that of Pierre Le Lune (16), suggesting that a “standard repertoire” was emerging for mid-century cookbooks. Notaker lists this volume under the heading *L'ecole des ragouts* rather than under the name of the author (2). The publisher, Jacques Canier and Fleury Martin in Lyon, had published an earlier version entitled *L'ecole des ragouts*, in which the name of La Varenne is nowhere to be seen. This is not surprising, since the text was derived from another (anonymous) work, *Le Cuisinier Methodique*, published in 1660 (17). The publisher then issued an additional version, designated as the 10th edition, in 1675, again lacking the authors name. However, in the 1680 edition, the same book carried the attribution: “*Par le Sieur de la Varenne.”* This, too, was clearly not the work of La Varenne and was reprinted from *Le Cuisinier Methodique* (18). It can only be surmised that the name of the author carried sufficient currency 30 years after his original publication for the publisher to feel the need to append his name inappropriately, an act that almost surely boosted sales to the general public and home cooks.

### Nicolas de Bonnefons, a Contemporary of La Varenne

In the first half of the seventeenth century, French cuisine was slowly moving from an avoidance of spices to an increased adoption of vegetables and more refined flavors. Emphases were placed on the specific attributes of vegetables and fruits, the maturity of poultry and animals, and the potential of butter and salt to enhance the taste of foods. The quality of produce was related to its cost and the seasons of the year, and use was therefore was linked to social status. New opportunities existed for access to a seasonal range of fresh produce throughout the year, including fruits and vegetables which ripened at different times of the year.

Into this slowly evolving world of culinary practice entered two important publications, *Le Jardinier Francois* (1651) and *Les Delices de la Campagne* (1654), authored by Nicolas de Bonnefons, a “valet de chamber” in the household of Louis XIV. The first was a book on gardening, reflecting the popularity of horticulture of the times, but also contained confectionary recipes. The more consequential follow-up volume, *Les Delices de la Campagne*, differed from other cookbooks in that it was organized into sections corresponding to specific products such as bread, wine, roots, fish, meat, poultry, etc., rather than according to sequential courses of a meal (2). Bonnefons opened the eyes of his readers to the pleasures of the countryside (19). He encapsulated the notion of allowing ingredients to express their natural tastes, rather than genuflecting to the medieval practice of smothering natural flavors by complex manipulations. *Le Jardinier Francais* (published in 1651, the same year as *Le Cuisinier Francois*) set the stage for an expectation that the informed cooks of the day appreciated the variety of plants and their seasonal specifics, their ripeness and readiness for the kitchen, and the physical properties of different species, which would dictate the most suitable technique of preparation and method of cooking. In this publication, Bonnefons laid out the knowledge of the gardener-cook. In *Le Delice de la Campagne*, he dealt with the interrelationship between ingredients, preparation techniques, and seasoning. For instance, it was not enough to know the species and age of the fowl or beast to be prepared (20). One also needed to know what parts of the animal imparted each nuance of flavor, or which differences in cooking time altered responsiveness to flavoring. All of these considerations went into the process of selection in the market, as did the region in France from which it originated, given that each specialty may have been sourced from a specific region. Milk, butter, cheese, foie gras, poultry, truffles, and even water required a knowledge of where and how they were resourced.

How should one compare the respective contributions of these talented cooks? La Varenne's initial intention was to direct his writings at culinary professionals, whereas Bonnefons aimed his at the masters and mistresses of wealthy households and at those who led a noble way of life. It is ironic that La Varenne's intention did not play out the way he intended it, since it is my contention that the durability of La Varenne's publication, through its multiple editions, could only have occurred if its buying public was very broad, involving all levels of society, and particularly the common citizens. In contrast, the very density and complexity of Bonnefons's writing style may have proved to be far too complex for those who simply wanted to get on with the job of cooking, thereby making his book less appealing to home cooks of distinguished households. For instance, Pinkard compares the number of words in a La Varenne recipe for a pigeon bisque (88 and 123 in the first and second editions, respectively) to six pages for the same dish by Bonnefons (4).

Both authors strived for purity of taste, unobscured by extraneous seasoning. La Varennes's recipes generally called for few ingredients, e.g., salt and water for fish dishes, and roux (emulsified butter, cream, and eggs) to season ragouts and fricassees. Bonnefons's dishes were spicier, sweeter, and more decorative, containing far more information than La Varenne's. Both used bread croutons as thickening agents and both, not surprisingly, clung to old favorites, subtly altered, which refused to die. Both cautioned about the excessive addition of acidic ingredients. Bonnefons was far more obsessive about sourcing, beginning in the garden, and was cognizant of seasonal difference for fruits, vegetable, poultry, and even water.

What accounted for the appearances of La Varenne's and Bonnefons's books at roughly the same time in Paris? The answer surely lies in the cultural and national emergence of Paris as one of Europe's most influential capitals. In the mid-1600s, France was the dominant power in Europe with the largest army, the largest economy, and a population three times larger than that of England. The arts were encouraged and supported by its ministers. The *Academie Francaise* was established a few decades before the publications of La Varenne's book and French was the language of the elite of many nations. When Louis XIV ascended to the throne and Versailles became symbol of his power, French cuisine emerged as the classic of the golden era.

French cuisine had been dominant for centuries and, whereas French cookbooks were translated into many European languages, no foreign cookbook was translated into French in the seventeenth or eighteenth centuries (3). French chefs were hired by the English nobility, with their cookbooks being translated and their recipes copied and adapted to local products and fashions. As pointed out above, French cuisine in the first half of the seventeenth century was slowly moving away from heavy and intrusive spicing to more subtle and less intrusive tastes, so the stage was set for an inflection point in culinary practice.

### Food Availability in Mid-Seventeenth Century France

Prior to the French revolution, the average caloric intake per person was about 2,000 a day. Peasants often sold their best produce and wheat while they themselves ate millet. They offered their eggs, kids, calves, and lambs for sale at the market while they ate salted pork. There was no comparison between what the common man consumed and the meat, poultry, game, cheeses, butter, olive oil, fruits, and vegetables available to the rich. In the sixteenth century, France emerged as the “homeland of fine fare,” receiving foods from the four corners of Europe, and leading to a culture of gourmandism and opulence.

To address the hypothesis that availability of specific foods in the French marketplace played a key role in La Varenne's choice of recipes, the following considerations are relevant. All are derived from Braudel (21).

#### Grains and Cereals

One of the most important constituents of the French diet of the time was grain. A hierarchy of qualities existed, determining which grains were affordable to each socio-economic group. Wheat was the most desirable, with the grain coming from the head of the corn being superior to that called “small corn,” was often mixed with the lesser cereals, i.e., rye, spelt, barley, and millet. The quality of bread made from these cereals was accordingly determined by the mixtures used. While there was a royal monopoly on grain in the latter years of the *ancien regime*, this was pushed out by private ownership.

For the common people there was a “monotony of diet,” which existed when carbohydrates exceeded about 60 percent of the dietary intake. More bread was consumed in the countryside than in towns and different grades of bread, e.g., with or without salt, or made from sifted or unsifted flour, virtually identified the social status of the buyer. Terms such as “*choyne*,” “*safleur*,” and “*reboulet*” defined these grades of bread. No more than four percent of the French population ate white bread; “*choyne*” was bread for the rich and “soft bread,” made with brewer's yeast, was the finest. At the time of La Varenne, bread was central to the diet, regardless of region or personal income.

#### Meat

Meats of all sorts were widely available in France in the sixteenth century, but after about 1550, fresh meat consumption was gradually replaced by cereals and smoked and salted meat, possibly in accordance with the current wisdom to “eat meat four times a day.” Cookbooks abounded with instructions about how to add spices such as ginger, cloves, nutmeg, thyme, basil and pepper to meats of all sorts, since these were reputed to drive off bloating and to “favor the seed.” La Varenne's inclusion of recipes for meat dishes was fairly limited.

#### Spices, Sugar, and Beverages

In the mid-seventeenth century the spice market was centered in Amsterdam. At this time, spices, which had been luxury ingredients, became widely consumed across Europe largely due to increased availability from the Far East. When prices began to fall and spices were no longer considered “exotic” additives, their desirability declined and they appeared less routinely on household tables. Aniseed, coriander, garlic, and pepper began to appear on the tables of the poor, while saffron remained a luxury ingredient.

Sugar was used widely in preparing all sorts of foods including meats, and what was previously a “medicine” began to be consumed as a necessary foodstuff. Strong spices were replaced by chocolate, alcohol, and tobacco as agents that enhanced the pleasure of a meal. Likewise, chocolate, tea, and coffee were all available in France prior to 1650.

#### Dairy Products

Cheese was a principal ingredient of the diet from mid-sixteenth century. France imported cheese, such as Gruyere, from Switzerland as a cheap source of protein. However, cookery books gave little coverage to cheese, with goat and ewe cheeses being regarded as inferior. Butter, available in European countries, was used widely in all sauces. Eggs were an everyday food.

#### Seafood

Religious rulings governed the consumption of fish; fast days, including Lent, prevailed during the reign of Louis XIV with consumption of eggs, meat, and poultry being prohibited at these times. Fresh fish and salted fish were permitted. Fish was obtained from the Atlantic coast, the English Channel, the North Sea, and the Baltic Sea. Sardines, anchovies, tunny, and herrings were in demand. Fresh water fish from the Loire included salmon and carp. Cod was eaten chiefly during Lent. Seafood is the predominant main course ingredient in *Le Cuisinier Francois*.

#### Vegetables

The fragile and transient nature of ripe vegetables and fruit made them prized ingredients among those who could afford them. They were sourced from specialty farmers but also from private gardens, which were becoming fashionable around the time of *Le Jardinier Francais* (22). Experienced gardeners tended the kitchen gardens of the wealthy, with talents that included the ability to raise vegetables and fruits out of season. By careful selection of different species and use of hot beds, it became possible to grow some species over most of the year.

### In the Kitchen and at the Table of the Home Cook

Well into the sixteenth century, it was not uncommon for one to rent a house if one wished to entertain guests over a meal. Tables were set for a predetermined number of guests with knives, spoons, plates, and goblets. Louis XIV forbade the use of forks, so fingers were the obvious option until forks entered common usage. Table manners had to wait for another century.

With the advent of a more “thoughtful” and refined cuisine (23), a home-dining culture evolved led initially by the elite, for whom polished manners at the table became an expectation if one were invited into the exclusive company at the table. The aristocracy set the tone of behavior expected of court society. These patterns spilled over into bourgeois society in France to become the new national norm. The evolution of this top-down diffusion of aristocratic refinement set France apart from German and English cooking styles, which were determined by populations whose tastes were determined largely by what was affordable.

While rank and prestige mattered, personal refinement, love of beauty, and a broad knowledge of the humanities and national affairs became the basis for conversation at the table. The dinner table became the setting for expression of values and ideas where forthright views and convivial discussions allowed for an equity of male and female views without a hierarchical seating plan.

Food courses in a typical meal were served *a la francaise*, meaning the simultaneous placement of multiple separate dishes on the table at the outset of the meal. This applied to family meals as well as to more formal occasions. Without the need for waiters or servants to serve individual courses, family and guests would partake of the offerings in no specific order. An obvious disadvantage of this approach was the loss of heat from warm dishes as the meal wore on, and their tastes suffered accordingly. By and large, the dishes devised and presented by La Varenne were not “fancy” in appearance and did not pander to the need for elaborate displays and visual effects. Instead, the main ingredient was complemented by understated sauces and garnishes, which did not obscure the fundamental taste of the dish.

Never to be denied their gustatory enjoyments, Parisians in the mid-seventeenth century wanted more than the savory dishes. They wanted something sweet and something soft, and so the pâtissier and the confectioner (*confiseur*) emerged, preparing pastries and confections in rooms separate from the main kitchen of the house.

### *Le Cuisinier Francois* Provides an Exposition of a New Cuisine

From the above it is apparent that the affordability of and access to fresh produce for the general population would skew selection to the use of grains, bread, dairy products, eggs, vegetables, fruit, and spices. The latter options were realistic, since spices were imported from the East, but the wide selection relegated the stronger ones such as pepper, ginger, cinnamon, and cloves to be used less, albeit not to their complete exclusion. Until the mid-seventeenth century, a wide range of ingredients was boiled in a common stockpot, which obscured their individual flavors, and then overloaded with spices (pepper, ginger, cinnamon, cloves) and acidic or sweet sauces. For example, in his 1615 cookbook, John Murrel called for sugar in 50% of savory recipes, cinnamon in 31%, ginger in 27%, and mace in 15% (24).

Apart from the notion of a healthy lifestyle in the countryside and provision of fruits and vegetables from gardens as promulgated by Bonnefons, La Varenne placed an emphasis on the harmony of identifiable flavors. His capon broth is an example where each of the variations on the recipe would be identifiable: “chicory slightly bitter, cardoon bitter and sweet, parsley fragrant and herbaceous” (4). His dishes almost never called for addition of sugar and rarely for ginger or cinnamon.

An important contribution of La Varenne was his attention to detail. For example, preparing to baste throughout the process was key to dishes such as suckling pig. The danger of overcooking fish was of importance, given the numerous fish dishes which dominate his book. Removal from the heat before deboning, adjusting the cooking temperature according to the size of the fish and the consistency of the flesh, and creating a court bouillon (the liquid in which a fish may be poached) were all steps which required attention to detail. No longer was the fire an appropriate source of heat since the temperature of sensitive ingredients (egg yolks) could only be controlled by raising or lowering the cooking pot, hardly an adequate means of achieving a delicate outcome. The elevated stove top, which included sections with burners set at different temperatures, allowed pots to be moved from one to another and enabled constant stirring, skimming, addition of ingredients, and deglazing, an essential development.

La Varenne's style of cooking was inventive, delicate, and played to the senses of smell and taste by delivering a variety and diversity of flavors and aromas [We have argued (25) that the sense of smell is an important activator of the digestive process prior to a morsel of food entering the mouth]. La Varenne clearly played to a broad sensorium. The result was the emergence of a cuisine that was delicate and refined, used only the juices of the principle ingredient as the basis for sauces and thickening agents, was augmented by light and subtle sauces, was based upon butter, cream, eggs, and flour, and highlighted, rather than obscure, natural flavors. Ingredients could be added at the table according to individual tastes instead of being considered essential ingredients.

Many of his dishes required little more than the juices remaining in the cooking pan. This approach was taken for roasts of birds, poultry, lamb, pork, rabbit, and other dishes. Bisques needed only bread croutons. Ragouts needed little more than wine or cream, or a roux of flour browned in butter. The resulting smoothness, coupled with the ability of fats (egg yolk) to enhance flavors, led to a transformation of French cuisine from being complex, spicy, acidic, or sweet to one that accentuated natural flavors, representing the refined and thoughtful style of a culinary practice.

Ragouts and potages were main elements in the repertoire of La Varenne. A ragout was a method of cooking meat, cut into pieces, browned and cooked without coloring, and with or without vegetables, in sauces that could augment their natural flavors. Such sauces no longer altered the viscosities and intensities that characterized medieval cuisine, which had used bread for this purpose. The potage was either a clear bisque or a category of thickened soup or stew in which meat, fish, or vegetables are boiled together with water to acquire a thickened consistency. La Varenne included many options for these for all days of the calendar.

Two methods innovated by La Varenne are worthy of attention: (a) emulsification and (b) the creation of a roux.

Emulsification was the process of maintaining fine droplets of oil or fat in a liquid, which assumed a creamy appearance and slightly viscous texture. The tendency of the two ingredients to separate required a stirring process and a precise ratio of one to the other. When mixed with the juices of meats or vegetables, a *jus* resulted (Modern French sauces, such as bearnaise or hollandaise are examples). Even gently heated cream or butter would serve this purpose, but more often egg yolk was the usual emulsifying agent. Buttery sauces became the staple of La Varenne for cooking fish, with only a small number of recipes requiring additional seasoning ingredients. Not only was a perception of smoothness imparted, but such sauces also added a glaze, which enhanced the visual appeal of the dish. The key to emulsified sauce was precision of temperature control. Addition of court-bouillon or some acid (vinegar, lemon juice) to cold beaten butter would make the sauce creamier. Adding remnants of meat by deglazing the cooking pan with wine or bouillon and the addition of butter or egg yolks would capture the essential flavor of the meat in an emulsified sauce. Such sauces also appear in La Varenne's recipes for vegetables (asparagus, cauliflower, artichokes) and seafood (lobster, langoustine). Where additional flavor was called for, non-intrusive additives such as chives, orange peel, and nutmeg could be added, and where an “edge” was called for, a dash of vinegar would do the trick.

A roux was created by sprinkling flour onto an ingredient (meat or vegetable) while it was being cooked. The emitted fats formed a paste with the flour and were augmented by the ingredient's juices to form a sauce, which was enhanced by further cooking. The advantage of this method was that the sauce, stabilized by the roux, allowed for advanced preparation and even for storage.

### The User-Friendly Layout of *Le Cuisinier Francois*

Fundamental to the design and organization of the cookbook, two key considerations seem to have been in the mind of La Varenne: the layout of the cookbook, which would best encourage its frequent perusal, and a selection of ingredients most likely to be available to its readers and users. His attention to both points may well explain the basis for the wide appeal of the book. It was organized around the Christian calendar, which differentiated between “lean days” (meals without meat), “flesh days” (meals with meat), and “fasting days,” with Lent being the longest period of fasting. Depending on the era, every week had at least 1 day of fasting, usually Friday. In the original Christian calendar there were between 150 and 250 lean days, meaning that unrestricted eating was permissible for only the remaining days. This practice became less restrictive in the sixteenth and seventeenth centuries. Meat and fats were clearly restricted as options for some days. Fish was less restricted and was included on lean days. Drying and smoking were also in common use. The creation of preserves was also described.

**Figure 3:**
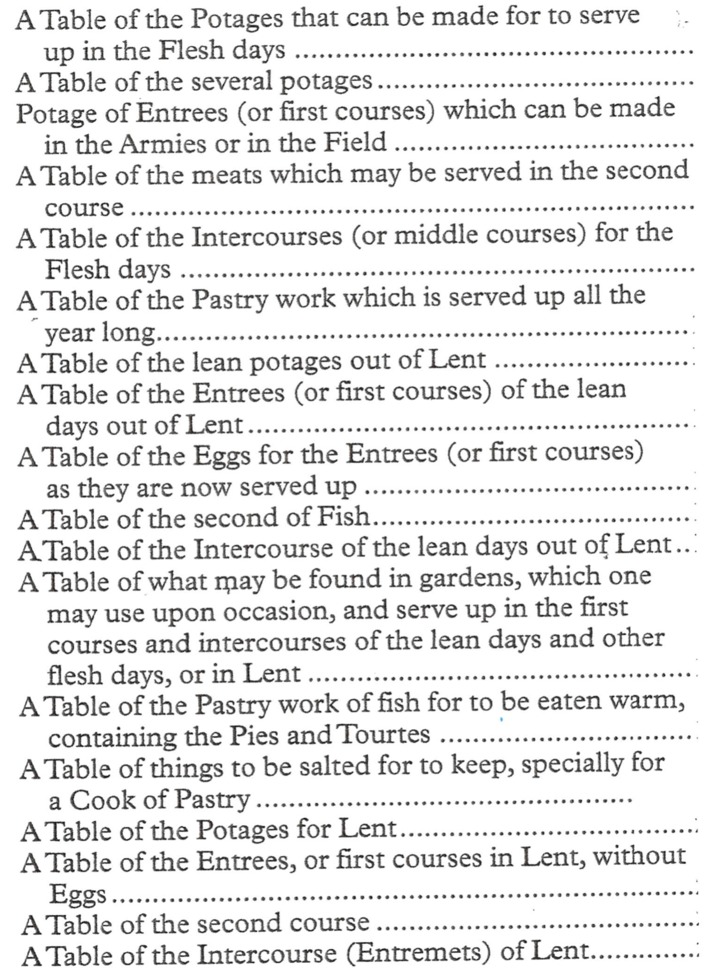
Table of contents modified from the English translationof the 1652 edition of *Le Cuisinier Francois* (7).

*Le Cuisinier* was thus organized into sections which provided menu options for flesh days, for lean days, for Lent days, and for pastries (savory), which could be eaten throughout the year (Figure 3). Included were potages, ragouts, gelees, roasts, farces, stews, rissoles, fritters, pates, pies, and fries. Also included were conserves and pickles.

Delicate flavors abounded. Sauces were made with butter and eggs. Ragouts were thickened with flour sautéed in fat. Subtle sauces, using butter and egg yolks as emulsifiers, have lived on to this day (e.g., hollandaise sauce). The initial assumption of La Varenne, who was expecting his readers to be the professional cooks well-versed in the basic techniques of cooking, may have been that lengthy and detailed descriptions were not called for. The brevity of his descriptions, however, may well have worked to his advantage in popularizing the book, in that inexperienced cooks were less likely to be intimidated by short instructions than having to wade through lengthy and tortuous recipes.

The majority of recipes in *Le Cuisinier Francois* were for seafood. Recipes for ~50 different species of fish are presented, the fish being mainly of the saltwater type and the most frequent being eel, pike, barbel, carp, salmon, monkfish, flounder, mackerel, and herring. These were the species most available in the markets at that time. There are only about 20 recipes for meat, including those for veal, mutton, beef, and pork, with a smaller number for poultry, pigeons, partridges, capons, and duck. As principal ingredients in recipes, there are only 15 for vegetables, and six for fruits included in the book.

## Conclusion

The success of *Le Cuisinier Francois* in 1651 is evidenced by the large number of editions published over the subsequent 50 years. The case is made that this could only have occurred if the book was purchased and used by the population at large rather than being restricted to professional cooks. This occurred against a backdrop of an almost complete absence of comparable publications in the prior half-century.

Explanations for this widespread and enduring success lie in La Varenne's precise and uncomplicated style of writing, his selection of principal ingredients within the reach of the household cook, and recipes that reflected the foods available to the French populace at the time of writing. Additionally, because *Le Cuisinier Francois* was laid out according to widely observed religious practices, finding the best options for the appropriate day of the month became an easy task for the home cook.

Although other cookbooks began to appear shortly after its publication in France and the rest of Europe, the inflection point undoubtedly occurred in mid-seventeenth century, arguably making La Varenne the most influential food writer of his time. His book initiated a departure from a “medieval” style of cooking in France, moving to one that was based upon natural flavors, a limited use of spices and uncomplicated cooking methods. This must have imparted a sense of empowerment and confidence to the home cook. In these terms, the author and his book were truly transformative.

## Author Contributions

LF originated the hypothesis, designed, and wrote the paper.

## Conflict of Interest

The author declares that the research was conducted in the absence of any commercial or financial relationships that could be construed as a potential conflict of interest.
